# Daily Probiotic Ayran Intake Reduces Gingival Inflammation: An Experimental Gingivitis Study

**DOI:** 10.3290/j.ohpd.b5784693

**Published:** 2024-10-14

**Authors:** Bahar Alkaya, Mehmet Cenk Haytac, Mustafa Özcan, Onur Uçak Türer, Hamza Gökhan Kayhan, Furkan Demirbilek, Wim Teughels

**Affiliations:** a Assistant Professor, Department of Periodontology Faculty of Dentistry, Cukurova University, Adana, Turkey. Data collection, critically revised the manuscript.; b Professor, Department of Periodontology Faculty of Dentistry, Cukurova University, Adana, Turkey. Study conception and design, critically revised the manuscript.; c Associate Professor, Department of Periodontology Faculty of Dentistry, Cukurova University, Adana, Turkey. Performed clinical treatment, critically revised the manuscript.; d Professor, Department of Periodontology Faculty of Dentistry, Cukurova University, Adana, Turkey. collected and analyzed data and prepared the manuscript, critically revised the manuscript.; e Research Assistant, Department of Periodontology, Cukurova University, Adana, Turkey. Collected and analysed data, prepared and critically revised the manuscript.; f Research Assistant, Department of Periodontology, Cukurova University, Adana, Turkey. Collected and analysed data, prepared and critically revised the manuscript.; g Professor, Department of Oral Health Sciences, KU Leuven & Dentistry (Periodontology), University Hospitals Leuven, Leuven, Belgium. Study conception and design, critically revised the manuscript.

**Keywords:** ayran, *Bifidobacterium bifidum*, dairy products, dental plaque, experimental gingivitis, *Lactobacillus acidophilus*, oral health, probiotics

## Abstract

**Purpose:**

This study investigates the effects of daily consumption of a probiotic ayran drink on gingival inflammation and the development of experimental gingivitis.

**Materials and Methods:**

This randomised, double-blind, placebo-controlled trial involved 54 volunteer students. The participants were randomly assigned to two groups: the control group received regular ayran for 42 days, while the test group received probiotic enriched ayran (including *Lactobacillus acidophilus* and *Bifidobacterium bifidum*) for 42 days twice a day. After 42 days, mechanical plaque control was stopped for 5 days. The plaque index (PI), gingival index (GI), probing bleeding (BOP), and probing depth (PPD) were measured at baseline, day 42, and day 47. At the same time, gingival crevicular fluid was taken for matrix metalloproteinase-8 examination.

**Results:**

The mean scores of BOP, GI, PI, and MMP-8 levels increases in both groups following the 5-day experimental gingivitis period compared to baseline and day 42. Patients using probiotic ayran had significantly less PI, GI, BOP scores and MPP-8 values (p = 0.002; p < 0.001; p < 0.001; p = 0.001; p = 0.001, respectively) at day 47 compared to the control group. No statistically significant differences in probing pocket depth (PPD) were observed at any time point.

**Conclusion:**

The present study suggests that daily consumption of a probiotic ayran drink containing *Lactobacillus acidophilus* and *Bifidobacterium bifidum* statistically significantly lowers clinical and immunological markers of gingival inflammation.

Gingivitis is the initial inflammatory reaction to the accumulation of dental biofilm.^44^ It is one of the most common diseases worldwide, affecting 90% of the population.^32^ Gingivitis is reversible, indicating that if the biofilm is eliminated, the condition disappears. However, in certain individuals, gingivitis can become chronic and advance to periodontitis.^54^ Gingivitis is considered a preliminary phase of periodontitis.^1^ Therefore, prevention, early diagnosis and treatment of gingivitis is very important to prevent periodontitis.^32^

Although self-administered oral hygiene is considered to be a critical component in maintaining periodontal health, the overall population does not consistently maintain good plaque control.^43^ The primary treatment for gingivitis therefore involves motivating and educating the patient to effectively remove dental plaque on a daily basis and professionally eliminate it by scaling.^4^ Mechanical biofilm management has some drawbacks including poor compliance, tendency to reacquire baseline biofilm level, restricted dexterity in some cases and lack of control of additional non-dental biofilms on the tongue or tonsils.^18^ In addition to mechanical biofilm removal to prevent periodontal disease, many antimicrobial agents are used both systemically and locally.^18,45^ However, since long-term use of these antimicrobials causes undesirable side effects, many alternative approaches are currently being explored.^5^ During the development of gingivitis, a significant change in the composition and function of the oral microbiota takes place; this is called dysbiosis.^6,15^ Dysbiosis refers to a condition that, among others, is characterised by a depletion of bacteria that are considered to be beneficial. This observation has underpinned the development of pro-, pre-, and synbiotic therapies. These therapies aim to restore the numbers of beneficial bacteria, thereby rebalancing the microbiota and promoting oral health. In recent years, the use of pro-, pre-, and synbiotics has emerged as a substitute for antibiotics and antiseptics.^14,58^

Many individuals are trying to change their immune systems and even accelerate the healing of periodontal tissues by using food supplements or so-called natural-food ingredients, such as fish oil/omega, fatty acids, protein-amino acid supplements, glucosamine and chondroitin sulfate, natural herbal products, and probiotics.^49^ The World Health Organization defines probiotics as living microorganisms that provide health benefits to the host when administered in adequate amounts.^23^ Probiotics have diverse applications, ranging from gastrointestinal health to oral health. Numerous studies in the literature have investigated the effects of probiotics on periodontal health and treatment.^37-39,45,52^ Probiotics have positive impacts on periodontal health by enhancing the quality of biofilm and regulating the inflammatory response of the host.^27^ Specific probiotic strains can stimulate and regulate various aspects of the innate and adaptive immune responses.^21^ Recently, much emphasis has been given to probiotic therapy and oral microbiota replacement therapy as ways to control periodontal disease.^40^ Furthermore, Jardini et al^25^ concluded that the use of *L. reuteri* in addition to subgingival instrumentation in the treatment of periodontitis in patients with type-2 diabetes mellitus may have important therapeutic results in the treatment of dyslipidaemia. The most effective probiotics for periodontal diseases belong to the *Lactobacillus* and *Bifidobacterium* genera.

The experimental gingivitis model, first described in 1965, is extensively utilised in studies investigating the pathogenesis of gingivitis.^34^ This model allows the induction of inflammatory responses under reproducible conditions, enabling the evaluation of antibacterial or anti-inflammatory agents.^16^ Recently, this model has been employed to assess the potential protective effects of probiotics as an adjunct therapy. Various types of probiotics and different delivery methods, such as milk^48^ and yogurt,^30^ have been shown to improve clinical gingival parameters. Additionally, these probiotic interventions have been associated with reductions in salivary periodontopathogens and inflammatory markers in gingival crevicular fluid (GCF).^30,48^ However, a study by Hallstrom et al,^22^ using the experimental gingivitis model, reported that lozenge-form probiotics did not yield significant improvements in gingivitis parameters.

Fermented milk and milk products possess considerable nutritional value and have the potential to alleviate health problems.^3^ Ayran is a salty, drinkable fermented dairy product that is popular in many countries globally and is high in calcium and vitamins.^47^ Shalabi et al^47^ investigated the nutritional and health benefits of ayran enriched with *Bifidobacterium animalis* ssp. *lactis BB-12*. Their study revealed a reduction in cholesterol levels and significant increases in the concentrations of monounsaturated and polyunsaturated fatty acids, including oleic acid, linoleic acid, and α-linolenic acid. Additionally, the antioxidant activity and folic acid content of the enriched ayran were notably enhanced. Based on these findings, they recommend the use of probiotic cultures to produce ayran with enhanced health benefits.^47^

Early periodontal disease diagnosis and therapy are possible with the help of biomarkers. GCF, saliva, and serum samples are used in biochemical and microbiological research. Assessing the accuracy of diagnostic molecular biomarkers in periodontitis patients, the GCF MMP-8 level was found to have good sensitivity and excellent specificity.^8^ Previous studies have shown that the MMP-8-saliva levels in periodontitis patients are >3-fold higher compared to healthy controls.^17^ Similarly, GCF MMP-8 levels correlated with clinical signs of disease activity in patients with chronic periodontitis.^8^ MMP-8 levels have been found to statistically significantly drop from baseline when a good response to periodontal therapy is determined.

The aim of this study was to investigate whether daily consumption of ayran enriched with the probiotics *Lactobacillus acidophilus* and *Bifidobacterium bifidum* can reduce plaque formation and gingival inflammation in healthy individuals, using an experimental gingivitis model. The null hypothesis posits that daily consumption of ayran enriched with these probiotics will not provide any additional benefits for periodontal health compared to regular ayran.

## MATERIALS AND METHODS

The study was conducted according to the Declaration of Helsinki, and the protocol was approved by the Ethics Committee of Cukurova University Faculty of Medicine (Project code 29-14). Prior to the study, the participants were provided with information regarding the study and were required to provide signed consent forms. The study is registered at clinicaltrials.gov (NCT: 06437925 ).

### Sample Size

G*Power, 3.1.9.7 (Heinrich Heine University, Düsseldorf, Germany) was used to determine the sample size. The primary outcome was PI. The sample size was calculated based on the effect size of the study conducted by Kuru et al.^30^ The study was powered to detect a minimum clinically significant difference of 0.80, alpha significance level of 0.05, and a power = %95. The minimum sample size was calculated as 52 patients.

### Study Protocol

This prospective, randomised, double-blind, and placebo-controlled study was conducted at the Department of Periodontology at Cukurova University between January and April 2023.

#### Inclusion criteria

- Systemically healthy subjects
- Subjects with gingivitis defined as a BOP sites ≥ 10% and PD ≤ 3 mm^11^
- No radiographic bone loss
- Non-smoking

#### Exclusion criteria

- History of using antibiotics or anti-inflammatory drugs or probiotic preparations or food supplements in the last 6 months
- Undergoing orthodontic treatment
- Active carious lesions
- Mouth breathing
- History of allergy to milk or fermented milk products.
- Taking medications affecting the gingiva and/or oral mucosa

155 university students were screened and a total of 54 volunteers were included in the study.

An oral and dental examination was performed for all selected participants. During the initial intervention on day 14, all participants underwent supragingival debridement (“Woodpecker Cavitron” ultrasonic device, Guilin Woodpecker Medical Instruments, Guangxi, China; Hu-Friedy scaler, Chicago, IL, USA) and received oral hygiene instructions (including interdental hygiene). All the participants received the same toothpaste and toothbrush (Colgate 360 optic white toothbrush, Colgate Total toothpaste; Colgate-Palmolive; Kocaeli, Turkey) and were instructed to brush according to the modified Bass technique. The patient was provided with dental floss (Curaprox; Kriens, Switzerland) or a suitable interdental brush (Curaprox), depending on the size and form of interdental spaces. At baseline (day 0: T1), the participants were randomly assigned to one of two groups.

- Probiotically enriched ayran (experimental group): 16 females and 11 males were included (mean age 22.48 ± 12 years).
- Regular ayran (control group): 9 females and 16 males were included (mean age 22.64 ± 1.4 years).

Participants in the control group used regular ayran (Eker; Bursa, Turkey), while participants in the experimental group used ayran enriched with *B. bifidum* and *L. acidophilus*. Both groups were instructed to drink the 195 ml of ayran twice a day (every 12 h) for 6 weeks. The probiotic enriched ayran contained ≥10^6^ colony-forming units (CFU)/g of *B. bifidum* and *L. acidophilus* strains, according to the manufacturer. Participants were recommended not to use other probiotic products during the study period and use the study products at 12-h intervals, as much as possible with no eating or toothbrushing for at least 1 h after consuming ayran. Participants performed standard oral hygiene procedures (twice-daily brushing and interdental cleaning) from baseline until the beginning of experimental gingivitis.

After 6 weeks (day 42: T2), an experimental gingivitis model was started with the discontinuation of all mechanical or chemical plaque control for 5 days in both groups. At the end of the study (day 47: T3, the end of experimental gingivitis), patients were given professional oral hygiene as well as topical fluoride, and received additional prophylaxis as needed.

### Clinical Measurements

**Fig 1:**
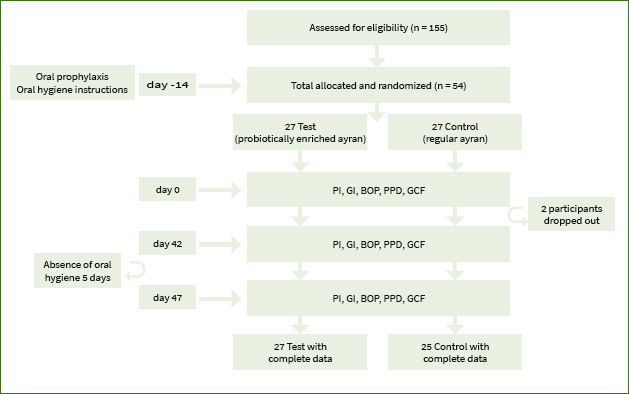
Study design.

The probing depth (PPD), gingival index (GI), plaque index (PI), bleeding on probing (BOP) and gingival crevicular fluid were recorded at baseline (T1), at the beginning of experimental gingivitis (T2), and at the end of experimental gingivitis (T3) (Fig 1).

For all oral clinical measurements, the North Carolina periodontal probe (PCP-UNC15; Hu-Friedy) was used. Periodontal measurements were performed at six sites (mesio-buccal, mid-buccal, disto-buccal, disto-lingual, mid-lingual and mesio-lingual) of each tooth. Mean scores were obtained by dividing the sum of all scores by the total number of surfaces evaluated.

PI was analysed as the primary outcome variable, while GI, BOP scores, PPD and level of MMP-8 were secondary outcome variables. PI was scored from 0 to 5 according to the colour change obtained with Mira-2 solution (Hager & Werken; Duisburg, Germany) using the modified Quigley & Hein Turesky index.^55^

GI was graded by visual assessment and mechanical stimulation of the gingival tissues, scoring the gingival condition according to the criteria defined by Löe and Silness.^33^

The bleeding-on-probing (BOP) index was determined by the presence/absence of bleeding 30 s after probing as described by van der Weijden et al.^56^

PPD was measured from the gingival margin to the base of the pocket, and rounded to the nearest millimeter.^19^

GCF was collected from the maxillary canine, right first premolar and mandibular canine teeth. Before collecting the gingival crevicular fluid, the existing plaque was carefully removed with sterile curettes. The tooth surfaces were dried and isolated with cotton rolls. Sterile paper strips (Oraflow; Hewlett, NY, USA) were placed in gingival pockets for 30 s. Blood-contaminated strips were discarded. All samples were frozen at -40°C until laboratory analysis. The total levels of MMP-8 were measured using a commercially available enzyme-linked immune sorbent assay (Picokine Elisa Kit, MyBioSource; San Diego, CA, USA) according to the manufacturer’s instructions. The MMP-8 level was determined in picograms/microleter (pg/µl).

### Randomisation

Randomisation was carried out by the study coordinator (MCH), who was not actively involved in the clinical trial, using a computer program (www.randomizer.org). Patients were enrolled in only one of the study groups, as each patient could only participate once in the study. Participants received probiotically enriched ayran or regular ayran twice daily during the study period. The type of ayran was masked by removing the paper covering the body of the container. The clinical treatments were performed by a blinded investigator (MO). In addition, the GCF sampling and the clinical parameters were measured by another blinded and calibrated examiner (BA). Examiner calibration was performed prior to the study on 10 subjects not included in the study to ensure reproducibility of clinical measurements. Measurements were repeated twice, 24 h apart. Inter-measurement correlation was found to be 96%.

### Statistical Analysis

IBM SPSS 25.0 (IBM; Armonk, NY, USA) was used for all statistical analysis of the data. Categorical variables were summarised as numbers and percentages, and continuous variables as mean and standard deviation (median and minimum-maximum, where appropriate). The chi-squared test was used for comparisons of categorical data. Shapiro-Wilk tests were used to confirm the normality of the distribution. Time-dependent changes were determined with the Friedman test. The difference between the measures was analysed using the Wilcoxon signed-rank test. The t-test was used between independent groups, Mann-Whitney U-tests were used for non-normally distributed parameters. A p-value < 0.05 was considered staitstically significant in all tests.

## RESULTS

The study included a total of 54 patients. Two participants in the control group who reported that they did not regularly use the study products were excluded from the study, resulting in a total of 52 participants (25 females/27males). Table 1 shows the demographic data of participants in each group. No statistically significant differences were found for age and gender distribution between groups (p>0.05). The participants reported no negative effects from the products they utilised.

**Table 1:** Demographic characteristics

	Probiotically enriched ayran (n = 27)	Regular ayran (n = 25)	p-value
	n (%)	n (%)	
Gender			
Female	16 (59.3)	9 (36)	0.093^‡^
Male	11 (40.7)	16 (64)	
	Meant ± SD (med)	Mean ± SD (med)	p-value
Age	22.48 ± 1.2 (23)	22.64 ± 1.4 (23)	0.771^†^

Statistical significance set at p < 0.05. †: Mann-Whitney U-test; ‡: chi-squared test.

The PI, GI, and BOP scores are presented in Table 2. The intragroup analysis showed no statistically significant differences for GI, PI. and BOP scores between baseline and day 42 (T2) for both groups, while all of these parameters were statistically significantly higher following the experimental gingivitis period (day 47: T3) compared to baseline and T2 (p < 0.05).

**Table 2:** Mean ( ± SD) PI, GI, and BOP outcome measures at day 0, 42, and 47 (D0, D42, D47)

Variable	Probiotically enriched ayran (n = 27) Mean ± SD (Med)	Regular ayran (n = 25) Mean ± SD (Med)	Inter-group comparison p^1^
**PI D0** ** D42** ** D47** **Intra-group comparison p2** **p3 (D0 vs D42)** **p4 (D0 vs D47)** **p5 (D42 vs D47)**	0.70 ± 0.11 (0.71) 0.73 ± 0.10 (0.74) 1.74 ± 0.54 (1.88) < 0.001* 0.211 < 0.001* < 0.001*	0.72 ± 0.13 (0.73) 0.75 ± 0.17 (0.78) 2.32 ± 0.53 (2.2) < 0.001* 0.538 < 0.001* < 0.001*	0.469^‡^ 0.369^†^ 0.002^†^*
**GI D0** ** D42** ** D47** **Intra-group comparison p^2^** **p3 (D0 vs D42)** **p4 (D0 vs D47)** **p5 (D42 vs D47)**	0.22 ± 0.07 (0.20) 0.23 ± 0.07 (0.20) 0.75 ± 0.24 (0.78) < 0.001* 0.129 < 0.001* < 0.001*	0.22 ± 0.07 (0.21) 0.23 ± 0.07 (0.21) 1.54 ± 0.2 (1.6) < 0.001* 0.698 < 0.001* < 0.001*	0.985^†^ 0.993^†^ < 0.001^†^*
**BOP % D0** ** D42** ** D47** **Intra-group comparison p^2^** **p^3^ (D0 vs D42)** **p^4^ (D0 vs D47)** **p^5^ (D42 vs D47)**	2.31 ± 0.89 (2.10) 2.33 ± 0.90 (2.10) 14.65 ± 3.80 (14.2) < 0.001* 0.094 < 0.001* < 0.001*	2.36 ± 0.64 (2.22) 2.45 ± 0.60 (2.33) 23.34 ± 5.99 (23.2) < 0.001* 0.132 < 0.001* < 0.001*	0.647^‡^ 0.415^‡^ < 0.001^†^*

p^1^: Mann-Whitney U-test; ‡: inter-group comprasions; p^2^ Friedman test, intra-group comprasions; p^3^-p^4^-p^5^ Wilcoxon signed-rank test, pair-wise comparisons; p^3^ comparing 0 vs D42; p^4^ comparing D0 vs D47; p^5^ comparing D42 vs D47. *p < 0.05

Similarly, the intergroup analysis showed that there were no statistically significant differences for PI, GI, and BOP scores between the probiotically enriched and regular groups at baseline and day 42. Following the 5-day experimental gingivitis period, the patients using probiotically enriched ayran had statistically significantly lower PI, GI and BOP scores (p = 0.002; p < 0.001; p < 0.001 respectively) at day 47.

The MMP-8 levels are presented in Table 3. The intra- and intergroup comparisons for MMP-8 again showed that while there were no inter- or intragroup differences up to day 42, the probiotically enriched ayran group presented statistically significantly lower MMP-8 levels compared to the controls.

**Table 3:** Mean ( ± SD) MMP-8 levels (pg/ µl), at 0, 42 and 47 days (D0, D42, D47)

Variable	Probiotically enriched ayran (n = 27) Mean ± SD (Med)	Regular ayran (n = 25) Mean ± SD (Med)	Inter-group comparison p^1^
**MMP-8 D0** ** D42** ** D47** **Intra-group comparison p^2^** **p^3^ (D0 vs D42)** **p^4^ (D0 vs D47)** **p^5^ (D42 vs D47)**	0.07 ± 0.03 (0.07) 0.07 ± 0.03 (0.07) 0.41 ± 0.14 (0.39) < 0.001* 0.310 < 0.001* < 0.001*	0.07 ± 0.03 (0.07) 0.07 ± 0.03 (0.07) 0.62 ± 0.22 (0.60) < 0.001* 0.484 < 0.001* < 0.001*	0.839^‡^ 0.912^‡^ 0.001^†^*

p^1^: Mann-Whitney U-test; ‡: inter-group comprasions; p^2^ Friedman test, intra-group comprasions; p^3^-p^4^-p^5^ Wilcoxon signed-rank test, pair-wise comparisons; p^3^ comparing 0 vs D42; p^4^ comparing D0 vs D47; p^5^ comparing D42 vs D47. *p < 0.05

PPD measurements showed no intra- or intergroup differences at any time points including 5 days after experimental gingivitis (p>0.05).

## DISCUSSION

This study evaluates the effects of daily consumption of ayran containing a probiotic combination of *Lactobacillus acidophilus* and *Bifidobacterium bifidum* on plaque development and gingival status in healthy individuals before and after experimental gingivitis induction. The results show that consuming ayran enriched with these 2 probiotic species reduces clinical and immunological markers of gingival inflammation following experimental gingivitis.

The evidence-based effects of probiotics on general health have led to more research in oral health. Even though the benefits of various probiotic microorganisms on periodontal health have been demonstrated, it is important to remember that the results obtained with probiotics cannot be generalised. The efficacy of these probiotics depends on their species, strain, dose, frequency of delivery, method of administration, and delivery vehicle.^57^ Therefore, conflicting findings exist on the efficacy of probiotics on periodontal health.^58^ The literature contains many studies on the effects of different probiotic *Lactobacillus* and *Bifidobacterium* probiotics strains on periodontal health. The effect of different *Bifidobacterium* species on oral health is controversial.^26^ Numerous studies in periodontology have used species such as *B. animalis* and *B. lactis*, demonstrating their efficacy such as reduced gingival and plaque indices as well as less gingival marginal bleeding.^24,30^ While the positive impact of *B. bifidum* on several health conditions, such as irritable bowel syndrome, diarrhea, and pathogen infections, has often been shown in laboratory and clinical trials, studies investigating the use of *B. bifidum* in periodontology are rare.^28^ *L. acidophilus* is one of the probiotic species frequently studied for oral health. Prevention of cariogenic activity of oral streptococci, antimicrobial, immune and inflammatory effects have been demonstrated in various studies.^9,10,51,59^

Studies are showing the potential benefits of using single-strain as well as multi-strain probiotic supplements.^7^ Toivanien et al^53^ used the probiotic combination of *Lactobacillus rhamnosus* and *Bifidobacterium* together, and found reduced plaque and gindival index scores in healthy individuals, but no probiotic-induced change in the microbial composition of saliva. Alanzi et al^2^ found that the probiotic combination of *Lactobacillus rhamnosus* and *Bifidobacterium lactis* improved gingival health in adolescents and reduced the microbial counts of *A. actinomycetemcomitans* and *P. gingivalis*. The present study was designed based on the theory that different species of probiotic bacteria can complement each other and enhance synergistic probiotic properties. To our knowledge, no previous studies have shown the effects of the combined use of *Lactobacillus acidophilus* and *Bifidobacterium bifidum* on gingival health.

Several studies have employed experimental gingivitis models to assess the efficacy of probiotics in oral health management. In the experimental gingivitis study by Kuru et al,^30^ the effect of consuming yogurt enriched with a probiotic *Bifidobacterium* species was compared to consuming conventional yogurt. Similar to our study, no differences were observed between groups in terms of GI, PI, BOP scores, GCF volume, and total IL-1β amount/concentration until day 28, the day that the experimental gingivitis development was initiated. However, five days after experimental gingivitis development, the probiotic yogurt group showed statistically significantly better clinical outcomes. Similarly, in the experimental gingivitis studies conducted by Slavik et al^48^ and Stab et al,^50^ groups consuming probiotic-containing milk achieved better clinical outcomes compared to control groups. In support of these studies, the present study showed that probiotic ayran consumption had a positive effect on GI, PI and BOP scores during experimental gingivitis development. In contrast, Halstrom et al^22^ and Olsen et al^35^ showed that the use of probiotics in experimental gingivitis patients made no statistically significant difference between the groups regarding clinical and immunological assessments. The reasons for the different results may be related to the use of different types of probiotics, different methods of administration, and different durations of probiotic use.

MMP-8, one of the MMPs found in the GCF of periodontitis patients, is an indicator of periodontal inflammation. It is the main collagenase involved in periodontal disease, and has the highest collagenolytic activity in GCF.^20^ It was reported that in people with chronic periodontitis, 80% of the collagenase in GCF samples is composed up of MMP-8.^8^ Neutrophils are the primary source of MMP-8 release, and they play a crucial role in the development of periodontal disease. In a study conducted by Ince et al,^24^ it was demonstrated that patients with chronic periodontitis who used *L. reuteri* lozenges for 3 weeks following scaling and root planing exhibited statistically significantly reduced levels of MMP-8, even after 180 days. Similarly, in the study by Kumar et al,^30^ a statistically significant decrease in MMP-8 levels was observed in chronic periodontitis patients using *L. reuteri* probiotics after non-surgical periodontal treatment. These findings are in accordance with the results of the current study, showing that probiotically enriched ayran consumption resulted in statistically significantly lower MMP-8 levels compared to controls after an experimental gingivitis period.

Dairy products have been shown to confer protective effects against tooth decay and periodontal disease, independent of their probiotic content.^13^ This protective function is attributed to compounds such as phosphorus, calcium, and proteins. Additionally, dairy products facilitate dentin remineralisation, inhibit the adhesion of pathogenic microorganisms to the tooth surface, and suppress the formation of pathogenic biofilms. They also serve as an effective delivery system for probiotic bacteria.^42^ Probiotic dairy products mainly studied in dentistry are milk, fermented milk, yogurt, kefir, and cheese.^13^ In the literature, emphasis is placed on the importance of regular consumption of probiotic dairy products to observe their effects on health.^37,47^ Manmontri et al^37^ have recommended daily consumption for stable and promising inhibition of pathogenic microorganisms. Oda et al^43^ investigated the effect of milk fermented with *Lacticaseibacillus rhamnosus* on the oral microbiota and the levels of four periodontal pathogens (*Tannerella forsythia, Porphyromonas gingivalis, Prevotella intermedia* and *Treponema denticola*) in a study on individuals with mental disabilities and gingivitis.^43^ The results of the study showed that probiotic products consumed daily suppressed the bacteria causing periodontal disease and were beneficial for mentally disabled individuals.^43^

Other factors such as the patient’s pretreatment gingival status and systemic conditions, their oral hygiene levels and smoking status may possibly influence the efficacy of oral probiotics in the treatment of plaque-induced gingivitis.^32^ In order to stardardise these factors in the current study, non-smoking students with similar age and oral hygiene levels and no systemic diseases were included. In addition, delivering the ayran products daily to the participants (2 bottles per day) directly by the study coordinator ensured control of their compliance.

In the literature, probiotic bacteria have been administered in various forms, including lozenges, toothpaste, yoghurt, and milk, with daily doses ranging from 1 to 4 times to day. However, the most common consumption pattern is twice daily. The duration of consumption can range from 21 to 84 days.^58^ In this study, ayran, a beverage that has not been previously examined as a means of probiotic administration, was administered twice daily for a period of 42 days, in accordance with the existing literature.

The precise cell count of a probiotic strain necessary in food to guarantee health benefits has not been defined. However, 10^6^ to 10^8^ CFU/g is thought to be a reasonable number to obtain probiotic benefits.^12^ In the present study, the manufacturer’s information stated a CFU/g of ≥10^6^, although the exact concentration was not known.

*Lactobacillus* and *Bifidobacterium* strains are generally regarded as safe for most people, no side effects were observed in the present study. However, there are some possible risks with their use such as taste disturbances, diarrhea, and allergic reactions.

### Limitations of the study

- The sample size, while statistically determined and sufficient, may not adequately represent the range of differences in the overall population, especially considering the homogeneous group of university students. Subsequent investigations should incorporate a broader range of participants in order to improve the applicability of the results.
- The study’s duration may not have been sufficient. Although our findings are adequate for examining immediate impacts, they may not accurately represent the prolonged advantages or possible negative consequences of longer consumption of probiotic ayran. Extended durations of observation are required to assess the long-term effects on periodontal health.
- The producer did not disclose the precise concentration and strains of the probiotic bacteria for commercial reasons.
- Regular diet of the participants: The consumption of other dairy food may have affected the results of the study.
- Blinding: Although an attempt was made to mask the type of ayran by removing the paper covering the body of the container, certain characteristics, such as taste, texture, or minor changes in the appearance of the containers may have inadvertently identied which group received probiotically enriched ayranx. Both patient and auditor blindness may have been impacted by this problem.

## CONCLUSION

Bearing the limitations of the study in mind, this study shows that consumption of probiotically enriched ayran with *Lactobacillus acidophilus* and *Bifidobacterium bifidum* reduces clinical and immunological markers of gingivitis following experimental gingivitis. This effect was seen at the clinical level (PI, GI, BOP) and for a GCF marker (MMP-8 levels). Probiotically enriched ayran may serve as a simple adjunct to standard oral care for promoting and maintaining oral health. It can be a reliable alternative to more expensive and difficult-to-access, over-the-counter probiotic food supplements. Clinical and microbiological research with more patients, longer durations, and different probiotics in varying dosages are required to demonstrate the longer-term effects of daily probiotic use on gingival health.
